# Acute bag-valve breathing maneuvers plus manual chest compression is safe during stable septic shock: a randomized clinical trial

**DOI:** 10.5935/0103-507X.20170004

**Published:** 2017

**Authors:** Clarissa Netto Blattner, Rafael Saldanha dos Santos, Fernando Suparregui Dias, Alexandre Simões Dias, Régis Gemerasca Mestriner, Silvia Regina Rios Vieira

**Affiliations:** 1 Faculdade de Enfermagem, Nutrição e Fisioterapia, Pontifícia Universidade Católica do Rio Grande do Sul - Porto Alegre (RS), Brasil.; 2 Hospital São Lucas, Pontifícia Universidade Católica do Rio Grande do Sul - Porto Alegre (RS), Brasil.; 3 Unidade de Terapia Intensiva, Hospital Pompeia - Caxias do Sul (RS), Brasil.; 4 Departamento de Fisioterapia, Hospital das Clínicas de Porto Alegre, Faculdade de Medicina, Universidade Federal do Rio Grande do Sul - Porto Alegre (RS), Brasil.; 5 Unidade de Terapia Intensiva, Hospital das Clínicas de Porto Alegre, Faculdade de Medicina, Universidade Federal do Rio Grande do Sul - Porto Alegre (RS), Brasil.

**Keywords:** Respiratory therapy, Breathing exercises, Shock, septic, Intensive care units, Airway management

## Abstract

**Objective:**

To evaluate the effects of bag-valve breathing maneuvers combined with standard manual chest compression techniques on safety, hemodynamics and oxygenation in stable septic shock patients.

**Design:**

A parallel, assessor-blinded, randomized trial of two groups. A computer-generated list of random numbers was prepared by an independent researcher to allocate treatments.

**Setting:**

The Intensive Care Unit at *Hospital São Lucas, Pontifícia Universidade Católica do Rio Grande do Sul*.

**Participants:**

Fifty-two subjects were assessed for eligibility, and 32 were included. All included subjects (n = 32) received the allocated intervention (n = 19 for the Experimental Group and n = 13 for the Control Group).

**Intervention:**

Twenty minutes of bag-valve breathing maneuvers combined with manual chest compression techniques (Experimental Group) or chest compression, as routinely used at our intensive care unit (Control Group). Follow-up was performed immediately after and at 30 minutes after the intervention.

**Main outcome measure:**

Mean artery pressure.

**Results:**

All included subjects completed the trial (N = 32). We found no relevant effects on mean artery pressure (p = 0.17), heart rate (p = 0.50) or mean pulmonary artery pressure (p = 0.89) after adjusting for subject age and weight. Both groups were identical regarding oxygen consumption after the data adjustment (p = 0.84). Peripheral oxygen saturation tended to increase over time in both groups (p = 0.05), and there was no significant association between cardiac output and venous oxygen saturation (p = 0.813). No clinical deterioration was observed.

**Conclusion:**

A single session of bag-valve breathing maneuvers combined with manual chest compression is hemodynamically safe for stable septic-shocked subjects over the short-term.

## Abstract (pt)

**Objetivo:**

Avaliar os efeitos de manobras de hiperinsuflação manual combinadas com pressão expiratória positiva associadas com técnicas padrão de compressão manual do tórax, com relação à segurança, à hemodinâmica e à oxigenação em pacientes com choque séptico estável.

**Delineamento:**

Este foi um estudo com dois grupos paralelos, randomizado e com avaliador cego. Um pesquisador independente preparou uma lista de randomização gerada por computador para alocação dos tratamentos.

**Local:**

Unidade de terapia intensiva do Hospital São Lucas, Pontifícia Universidade Católica do Rio Grande do Sul.

**Participantes:**

Foram avaliados 52 indivíduos quanto à elegibilidade e, destes, 32 pacientes foram incluídos no estudo. Todos os indivíduos incluídos (n = 32) receberam a intervenção alocada (n = 19 para o Grupo Experimental e n = 13 para o Grupo Controle).

**Intervenção:**

Vinte minutos de manobras de hiperinsuflação manual combinadas com pressão expiratória positiva associadas com técnicas padrão de compressão manual do tórax (Grupo Experimental) ou compressão do tórax, conforme o uso rotineiro de nossa unidade de terapia intensiva (Grupo Controle). Foi realizado seguimento imediatamente após e 30 minutos após a intervenção.

**Principal métrica de desfecho:**

Pressão arterial média.

**Resultados:**

Todos os indivíduos inscritos concluíram o estudo (n = 32). Não identificamos efeitos relevantes na pressão arterial média (p = 0,17), frequência cardíaca (p = 0,50) ou pressão média da artéria pulmonar (p = 0,89) após ajuste quanto à idade e ao peso do participante. Após ajuste dos dados, ambos os grupos foram idênticos com relação ao consumo de oxigênio (p = 0,84). A saturação periférica de oxigênio tendeu a aumentar com o tempo em ambos os grupos (p = 0,05), e não ocorreu associação significante entre o débito cardíaco e a saturação venosa de oxigênio (p = 0,813). Não se observou deterioração clínica.

**Conclusão:**

Uma única sessão de manobras de hiperinsuflação manual combinadas com pressão expiratória positiva associadas com técnicas padrão de compressão manual do tórax é hemodinamicamente segura em curto prazo para pacientes com choque séptico estável.

## INTRODUCTION

Sepsis is a continuum of events that are triggered by serious infection.^(1)^ The interaction between pro-inflammatory, anti-inflammatory and apoptotic mediators leads to circulatory failure, myocardial depression, increased metabolic rate and abnormalities in the oxygen demand/reserve ratio, contributing to global tissue hypoxia.^(2,3)^ Therefore, hemodynamic changes must be strictly monitored to minimize clinical complications.^(4)^

Currently, physiotherapy is widely used in intensive care units (ICUs) because it has positive effects in critically ill patients. These benefits may result from the physiological effects of early mobilization and improved clearance of bronchial secretions.^(5-7)^ Moreover, a previous Brazilian trial showed increased oxygen consumption (VO_2_) and decreased venous oxygen saturation (SvO_2_) due to an increase in the oxygen extraction rate (ERO_2_) after early mobilization in critically ill patients.^(8)^

However, animal studies have shown that manual hyperinflation leads to harmful effects, such as reduced cardiac output, compensatory vasoconstriction and increased systemic vascular resistance.^(9)^ Thus, hemodynamic changes that are inherent to the procedure might contraindicate chest physiotherapy in some clinical conditions, such as septic shock.

To address this issue, our research group recently evaluated the acute effects of chest manual compression techniques on hemodynamics, inflammatory profile and oxidative stress in septic shock patients; in the study, we observed increased oxygenation as well as reduced lactate levels and oxidative stress, with no changes in hemodynamics.^(10)^ However, it remains unclear whether vigorous chest physiotherapy techniques, such as acute bag-valve breathing maneuvers combined with manual chest compression, affect hemodynamics and oxygenation in septic-shock patients.

The research questions asked were the following: What are the short-term effects of acute bag-valve breathing maneuvers (manual hyperinflation associated with positive end-expiratory pressure valve) combined with standard manual chest compression techniques on hemodynamics and oxygenation in stable septic-shock subjects? Is this procedure clinically safe over the short term?

The aim this study to evaluate the effects of bag-valve breathing maneuvers combined with standard manual chest compression techniques on safety, hemodynamics and oxygenation in stable septic shock patients.

## METHODS

This study is a parallel, assessor-blinded, randomized trial of two groups (Experimental and Control Groups). Participants were recruited from the ICU. Outcomes were measured before, immediately after and at 30 minutes after the intervention. Because the registration of clinical trials has become mandatory in Brazil since 2012, the current study was registered retrospectively in The Brazilian Clinical Trials Registry under the number RBR-283ZTS. The *Pontifícia Universidade Católica do Rio Grande do Sul* Ethics Committee approved this study. All participants gave their written informed consent before data collection began.

**Figure 1:**
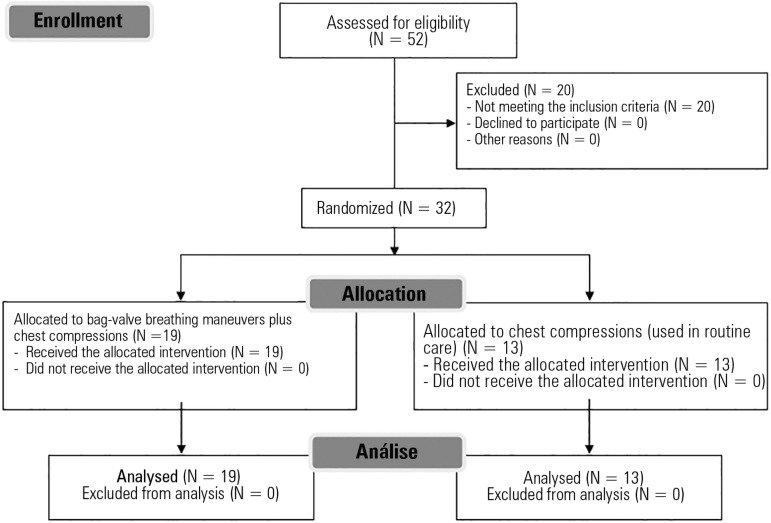
Study flow chart.

Eligible participants included all adults aged between 19 and 80 years with septic shock, using a pulmonary artery catheter (Swan-Ganz), and receiving mechanical ventilation. The exclusion criteria were pregnancy; acute myocardial infarction occurring less than three months before the study; previous chronic pulmonary disease; severely ill cardiac disease (heart ejection fraction < 30%); life expectancy of less than 24 hours; and impossibility of the family member or guardian signing the free, informed consent form. Overall, 52 subjects were assessed for eligibility, and 32 were included (Figure 1). The study took place at the ICU of the *Hospital São Lucas*, Porto Alegre, Brazil from August 2009 to February 2013. *Hospital São Lucas* is a reference hospital for sepsis treatment in South Brazil.

### Intervention

Patients were randomly assigned to receive bag-breathing maneuvers combined with chest compression (Experimental Group) or standard routine care (Control Group). The Experimental Group received 20 minutes of bag-valve breathing maneuvers combined with chest compression. A spring-loaded valve was used to maintain the positive end-expiratory pressure at 10cmH_2_O. A 3L self-inflating bag (AMBU) connected to a flow of 10L/min was used to deliver an inspiratory pressure of 40cmH_2_O. A manometer was coupled to the system to control the delivered pressures. Long breaths or alternations of rapid and slow manual hyperinflation were used as breathing maneuvers. Breathing rate during the intervention ranged from 18 to 30 breathing cycles per minute (bpm). Inspiratory time and inspiratory hold varied among the subjects. The endotracheal tube was suctioned immediately after the intervention using a closed-suction system (Trach Care, TM).

Control Group subjects received 20 minutes of physiotherapy according to the hospital's standard routine care (chest vibrocompression, passive manual expiratory therapy and compression-decompression maneuvers, promoting bronchial clearing and pulmonary re-expansion). The endotracheal tube was suctioned with a similar system and for an equivalent length of time to that applied in the Experimental Group.

All subjects remained in dorsal decubitus with the head of the bed at 40° to avoid bias during data collection. Mechanical ventilation values were adjusted in both groups according to the ICU's routine: controlled volume setting, a tidal volume of 6 - 7mL/kg, and a fractional inspired oxygen concentration (FiO_2_) of 0.45 - 0.8.

For ethical reasons, we did not evaluate an additional Control Group (without physiotherapy care). Critically ill subjects usually receive daily chest compression as standard care in this ICU.

### Outcome measures

In the current study, we tested the hypothesis that bag-valve breathing maneuvers (manual hyperinflation associated with positive end-expiratory pressure valve) plus manual chest compression would (1) not induce deleterious effects on hemodynamics and oxygenation in stable septic shock subjects and (2) be clinically safe over the short term, considering hemodynamics. The primary outcome was mean arterial pressure (MAP). Secondary outcomes were heart rate (HR); mean pulmonary artery pressure; pulmonary vascular resistance index; cardiac index; right ventricular ejection fraction; end diastolic volume index; arterial oxygen saturation (SpO_2_); central venous oxygen saturation (ScvO_2_); oxygen consumption rate (VO_2_); oxygen extraction rate; and oxygen delivery rate. All endpoints were measured before, immediately after, and 30 minutes after the intervention.

### Randomization

Participants were randomly assigned following simple randomization procedures (based on computerized random numbers) to receive the experimental or control procedures. The computer-generated list of random numbers was prepared by an independent researcher. The allocation sequence was concealed from the researcher who enrolled and assessed the participants in sequentially numbered, opaque, sealed and stapled envelopes. After obtaining the patient's consent, the researcher called a contact who was independent of the recruitment process for allocation consignment.

### Blinding

The patients, physicians and physiotherapists were aware of the patient's allocation (to the Experimental or Control Group), and the outcome assessors and data analysts were kept blinded to the allocation.

### Data analysis

Sample size was calculated using previously published data from an animal model.^(9)^ These data were chosen because, to the best of our knowledge, the study from which it came was the most similar to ours at the time of the study design. Heart rate, MAP, and pulmonary artery pressure were used as the main endpoints. Twenty-six subjects were required (13 in each group) for a significance level of 5% and power of 85%, with one standard deviation as the expected effect size. Six extra subjects were recruited to allow for the possibility of losses or dropouts. Normally distributed data were expressed as the means ± standard deviation. Qualitative endpoints were described using absolute and relative frequencies. To evaluate group homogeneity before the intervention, the independent Student *t*-test or the Mann-Whitney test were applied, depending on the data profile. Pearson's Chi-square test was applied to compare qualitative parameters between the groups. Analysis of Variance (ANOVA) or repeated measures ANOVA were used for inter and intragroup comparisons. Bonferroni post hoc tests were performed as indicated. Analysis of Covariance (ANCOVA) for repeated measures was used to control for confounding factors. The results were considered significant when p ≤ 0.05. SPSS 18.0 (Statistical Package for the Social Sciences, Inc., Chicago, USA) was used to analyze the data.

## RESULTS

### Flow of participants, therapists, and centers through the study

Recruitment and data collection were carried out between August 2009 to February 2013.

Overall, 52 subjects were assessed for eligibility, of which 32 were included. Nineteen subjects were allocated to the Experimental Group, and 13 were allocated to the Control Group. No complications occurred during the control or the intervention procedures. All included subjects (n = 32) received the allocated intervention (n = 19 for the Experimental Group; n = 13 for the Control Group) and were included in the data analysis. The baseline characteristics of the subjects are shown in table 1.

**Table 1:** General characteristics of the patients

Variables	Experimental Group (N = 19)	Control Group (N = 13)	p value
Age	46.4 ± 18.6	61.2 ± 15.4	0.025
Weight	75.6 ± 12.3	86.7 ± 15.9	0.034
Height	170.5 ± 8.6	171.2 ± 11.9	0.863
Sex			0.770
Male	8 (42.1)	7 (53.8)	
Feminino	11 (57.9)	6 (46.2)	
Mechanical ventilation duration	7.00 ± 4.48	6.31 ± 2.63	0.587
Type of sepsis			0.261
Urinary	4 (21.1)	5 (38.5)	
Pulmonary	10 (52.6)	3 (23.1)	
Abdominal	4 (21.1)	5 (38.5)	
Liver	1 (5.3)	0 (0.0)	
SOFA	13.61 ± 4.7	14.21 ± 3.9	0.210
Vasopressor (mc/kg/minuto)	0.20 ± 0.09	0.22 ± 0.09	0.647
Death	10 (52.6)	9 (69.2)	0.567

SOFA - Sequential Organ Failure Assessment Score. The results are expressed as number (%) and means ± standard deviation.

The subjects were similar in all variables between groups, except for age (p = 0.025) and weight (p = 0.034). The intervention group consisted of younger subjects who weighed less. Repeated measures ANCOVA was performed to control for any sample bias (adjustments for age and weight factors). As expected, no significant differences were detected after ANCOVA adjustment.

Mean arterial pressure, the primary outcome, did not significantly differ (p > 0.05) between the Experimental (baseline: 84.0 ± 9.3mmHg; 30 minutes later: 84.7 ± 12.8mmHg) and Control (baseline: 76.7 ± 14.1mmHg; 30 minutes later: 78.8 ± 13.0mmHg) Groups. Because the groups did not significantly differ, an effect size was not calculated. No adverse events or side effects occurred. This finding was expected the because hemodynamic changes induced by septic shock and the Swan-Ganz catheter indication are usually independent of age and body mass index. We found differences in MAP when testing "group main effects" (adjusted for age and weight) (p = 0.029). However, there were no additional time-point main effects (before, immediately after, or at 30 min after the intervention) (p = 0.647) or "time-point" and "group" interaction effects (p = 0.318), suggesting this MAP difference was unrelated to the experimental procedures. The baseline values of oxygen saturation (SaO_2_) (94.2 ± 3.6), HR (106.1 ± 13.7), MAP (84.0 ± 9.3) and VO_2_ (146.0 ± 70.3) showed that the subjects were hemodynamically stable at baseline.

### Hemodynamic impact of the procedures

The intragroup comparison (repeated measures) is shown in table 2. We found a hyperacute "time effect" in HR, cardiac output, MAP and mean pulmonary artery pressure values. However, these differences were not found to be significant when the data were adjusted for subject age and weight (Table 2). Subtle differences are expected because suctioning usually impacts hemodynamics acutely. In addition, there was a subtle transient rise in MAP after bag valve maneuver, which returned to values similar to those at baseline and those of the control group within 30 minutes. No clinically relevant changes were observed in the analyzed data.

**Table 2:** Hemodynamic variables according to group and moment evaluated

Variables	Experimental Group (N = 19)			Control Group (N = 13)		
	Before	Immediately after	30 min after	Before	Immediately after	30 min after
Heart rate (bpm)	106.1 ± 13.7	113.8 ± 17.6	109.7 ± 15.0	109.3 ± 20.0	113.4 ± 22.3	106.9 ± 19.2
Cardiac output (L/min)	5.76 ± 1.16	5.28 ± 0.91*	5.15 ± 0.79	5.66 ± 1.26	5.20 ± 0.84	5.09 ± 0.64
MAP (mmHg)	84.0 ± 9.3	91.8 ± 12.8*	84.7 ± 12.8	76.7 ± 14.1	80.4 ± 15.5	78.8 ± 13.0
MPAP (mmHg)	27.9 ± 3.7	28.8 ± 4.7†	27,1 ± 3,4	29.7 ± 7.7	30.9 ± 7.3	29.5 ± 6.5
PVRI (dy•sec/cm^5^/m^2^)	469.9 ± 272.8	503.4 ± 275.3	460.9 ± 285.7	526.6 ± 261.7	612.9 ± 293.5	566.0 ± 270.7
Cardiac index (L/minuto/m^2^)	2.53 ± 0.98	2.37 ± 1.10	2.51 ± 1.18	2.41 ± 1.13	2.28 ± 1.27	2.54 ± 1.19
RVEF (%)	24.1 ± 8.5	22.5 ± 9.0	24.0 ± 8.3	26.9 ± 11.1	24.9 ± 11.5	27.4 ± 11.8
EDVI (mL/m^2^)	112.1 ± 40.3	109.2 ± 32.7	115.4 ± 44.4	93.8 ± 31.9	94.2 ± 33.2	93.4 ± 30.2

MAP - mean arterial pressure; MPAP - pulmonary mean arterial pressure; PVRI - pulmonary vascular resistance index; RVEF - right ventricular ejection fraction; EDVI - end diastolic volume index. Values expressed as the means ± standard deviation; min: minutes. Within-group effects at

* p < 0.001 and

† p < 0.03.

### Oxygenation impact of the procedures

Oxygenation data are shown in table 3. There was an isolated "time effect" on SAO_2_ (p < 0.001), which remained significant after ANCOVA adjustments (for age and weight, p = 0.05). This finding showed that oxygen saturation tended to increase over time in both groups. However, no differences between groups were found (p > 0.05). Our data showed a significant increase in VO_2_ at 30 minutes after the intervention (p = 0.01). No differences were found in the Control Group (p = 0.39). However, these effects were not significant after ANCOVA adjustments for age and weight (Table 3). Moreover, no significant association was found between cardiac output and ScvO_2_ (p = 0.813).

**Table 3:** Oxygenation variables according to group and moment evaluated

Variables	Intervention Group (N = 19)			Control Group (N =13)		
	Before	Immediately after	30 min after	Before	Immediately after	30 min after
SaO_2_	94.2 ± 3.6	94.2 ± 3.6*	97.6 ± 2.1	94.5 ± 3.8	96.1 ± 3.9	97.1 ± 2.4
SvO_2_	69.8 ± 7.5	71.8 ± 9.8	75.7 ± 7.8	72.7 ± 12.0	72.7 ± 12.0	71.9 ± 8.3
VO_2_	146.0 ± 70.3	147.1 ± 70.8†	165.9 ± 72.9	158.1 ± 101.8	145.9 ± 60.8	156.1 ± 56.7
ERO_2_	24.1 ± 6.6	25.8 ± 7.9	26.2 ± 9.8	24.1 ± 11.6	26.5 ± 8.9	27.0 ± 8.5
DO_2_	512.8 ± 120.3	536.3 ± 119.7	538.2 ± 120.3	516.2 ± 195.2	492.2 ± 141.6	491.7 ± 135.4

SaO_2_ - arterial oxygen saturation; SvO_2_ - venous oxygen saturation; VO_2_ - oxygen consumption rate; ERO_2_ - oxygen extraction rate; DO_2_ - oxygen delivery rate; min - minutes. Values expressed  as the means ± standard deviation.

* p < 0.001;

† p = 0.030.

## DISCUSSION

Physiotherapy has been shown to have several benefits for critically ill patients. Bronchial clearance, the prevention and resolution of atelectasis, increased gas exchange, and improved inspiratory muscle performance are examples of physiotherapy goals in ICUs.^(11-14)^ This study was conducted in an ICU in which all mechanically ventilated patients usually receive respiratory and motor physiotherapy procedures three times per day.

This study showed that both procedures, chest compression or bag-valve breathing maneuvers combined with chest compression, tend to increase SaO_2_ in stable septic-shock subjects without short-term clinically relevant changes in hemodynamics. Although our main goal was to test acute hemodynamics safety, the lack of differences in the results obtained between the two techniques is important to highlight. This interesting finding may be explained by two hypotheses. First, to find clinically relevant differences between the used chest physiotherapy techniques after applying both in a single session is unlikely.^(7,11)^ Second, while previous studies have suggested that chest physiotherapy induces beneficial effects on airway clearance, preventing ventilation-associated pneumonia, pulmonary complacence and resistance,^(15-18)^ the studied protocols applied a wide combination of techniques. Thus, the clinical benefits of chest physiotherapy might be provided when using techniques in combination.^(11)^ Regardless, it seems premature to draw conclusions regarding the clinical effectiveness of the studied techniques based only on the current trial data. Further research is needed to clarify the previously mentioned hypotheses.

Hemodynamic monitoring has been the subject of studies in critical care research.^(4,9,19)^ A previous randomized clinical trial has shown that the use of a positive expiratory pressure (PEP) mask can significantly increase mean arterial pressure, mean pulmonary artery pressure, central venous pressure and pulmonary artery occlusion pressure. However, these differences were considered not to have a relevant harmful clinical impact on hemodynamic stability. Indeed, positive expiratory pressure might provide several benefits to patients, such as lung re-expansion and airway clearance optimization.^(20,21)^ In similar studies, increased PaO_2_ and SaO_2_ with decreased PaCO_2_ were observed. An improvement in respiratory mechanics and bronchial clearance was correlated with this effect.^(10)^

Our findings are in agreement with these studies. We showed that the bag-valve breathing maneuvers combined with manual chest compression induced no relevant hemodynamic changes or clinical deterioration. The subtle hyperacute differences that were observed immediately after the control and intervention procedures were probably similar to those observed during routine standard care, i.e., airway suctioning, decubitus change or body hygiene care. Although we tested the effects of the bag-valve breathing maneuver on acute hemodynamics safety, a possible procedure-related secondary lung injury^(16)^ was not assessed. However, we adopt acute bag-breathing maneuvers only up to 40cmH_2_O (manometer-controlled) as a protective strategy to avoid lung injury as suggested in the literature.^(15)^ This study limitation represents a possible subject for further investigation. Cardiovascular complications in sepsis are associated with poor outcome.^(1)^ In septic shock, the oxygen consumption/delivery rate is critical. Thus, hemodynamic profile should be strictly controlled to support patient metabolic demand.^(22)^

Currently, ScvO_2_ is the gold standard measurement for assessing the balance between global oxygen supply and demand, which is correlated with cardiac output. Low SvO_2_ is a strong biomarker for cardiac output insufficiency. However, normal cardiac output values do not indicate that the oxygen supply adequately meets oxygen tissue demand.^(23)^ For example, the subjects enrolled in this study presented higher baseline cardiac output values than normal, a finding that reinforces previously published data.

Septic patients have a high VO_2_ and are highly dependent on its supply. The increase in VO_2_ associated with the reduction in oxygen extraction through the peripheral tissues can impair the microcirculation and result in tissue hypoxia. In addition, the diminished venoarterial difference suggests that oxygen is not able to reach the peripheral tissues.^(24)^

Our results showed that oxygen saturation tended to increase over time in both groups in agreement with the literature.^(25)^ Moreover, we found no important effects on VO_2_ when the data were adjusted for age and weight. This finding suggests that short-term physiotherapy effects are unrelated to harmful VO_2_ increases.^(24)^ Furthermore, bag-valve breathing maneuvers did not promote additional benefits over the short term when compared to conventional chest physiotherapy. Because only one session was applied, further trials are necessary to clarify the long-term effects of bag-breathing maneuvers on hemodynamics safety as well as its therapeutic effects.

This study has several limitations. The Swan-Ganz catheter, an invasive device for monitoring, provides accurate hemodynamics data.^(26)^ Contrariwise, to the best of our knowledge, few studies have used this catheter to assess the effects of physiotherapeutic techniques, a fact that can be explained by the restricted medical indication of the Swan-Ganz catheter. Paradoxically, while the catheter provides high-quality data, it is important to consider limitations in its external validity. For example, our ICU cares for an average of 12 Swan-Ganz monitored subjects every year. Therefore, our findings cannot be extrapolated to all critically ill patients.

Furthermore, the inclusion of subjects with distinct septic foci might have resulted in selection bias. In addition, metabolic parameters, such as plasmatic lactate and calorimetric measures, were not assessed. These endpoints could be the subject of further clinical trials that are designed to clarify the biochemical mechanisms that are related to chest physiotherapy procedures.

Because the included septic-shocked subjects were critically ill,^(1)^ secondary measurements (i.e., mortality rate, number of days under mechanical ventilation, length of stay in the ICU and ventilator-associated pneumonia) were highly influenced by organ dysfunction/failure (data not shown). Thus, it was impossible to establish the statistical weight (contribution) of the physiotherapy techniques (applied in a single session) on hard outcomes using the present sample size. Further trials are needed to elucidate this point.

## CONCLUSION

In conclusion, a combination of acute bag-valve breathing maneuvers with chest compression techniques was safe and had no deleterious hemodynamic effects over the short term. Subtle differences at baseline were not clinically relevant. Finally, oxygen saturation tended to increase over time in both groups, demonstrating that the evaluated techniques produced a significant benefit. Long-term trials are required to elucidate the size effect and biochemical mechanisms of different chest physiotherapy protocols in septic-shocked subjects.

Overall, we conclude that a single session of bag-valve breathing maneuvers combined with manual chest compression is hemodynamically safe for stable septic-shocked subjects over the short term. However, no acute benefits were observed compared to the usual care given.
